# Glucolipotoxicity: A Proposed Etiology for Wooden Breast and Related Myopathies in Commercial Broiler Chickens

**DOI:** 10.3389/fphys.2020.00169

**Published:** 2020-03-13

**Authors:** Juniper A. Lake, Behnam Abasht

**Affiliations:** ^1^Center for Bioinformatics and Computational Biology, University of Delaware, Newark, DE, United States; ^2^Department of Animal and Food Sciences, University of Delaware, Newark, DE, United States

**Keywords:** wooden breast, white striping, broiler, myopathy, pectoralis major, diabetes, spaghetti meat, dorsal cranial myopathy

## Abstract

Wooden breast is one of several myopathies of fast-growing commercial broilers that has emerged as a consequence of intensive selection practices in the poultry breeding industry. Despite the substantial economic burden presented to broiler producers worldwide by wooden breast and related muscle disorders such as white striping, the genetic and etiological underpinnings of these diseases are still poorly understood. Here we propose a new hypothesis on the primary causes of wooden breast that implicates dysregulation of lipid and glucose metabolism. Our hypothesis addresses recent findings that have suggested etiologic similarities between wooden breast and type 2 diabetes despite their phenotypic disparities. Unlike in mammals, dysregulation of lipid and glucose metabolism is not accompanied by an increase in plasma glucose levels but generates a unique skeletal muscle phenotype, i.e., wooden breast, in chickens. We hypothesize that these phenotypic disparities result from a major difference in skeletal muscle glucose transport between birds and mammals, and that the wooden breast phenotype most closely resembles complications of diabetes in smooth and cardiac muscle of mammals. Additional basic research on wooden breast and related muscle disorders in commercial broiler chickens is necessary and can be informative for poultry breeding and production as well as for human health and disease. To inform future studies, this paper reviews the current biological knowledge of wooden breast, outlines the major steps in its proposed pathogenesis, and examines how selection for production traits may have contributed to its prevalence.

## Introduction

**FIGURE 1:**
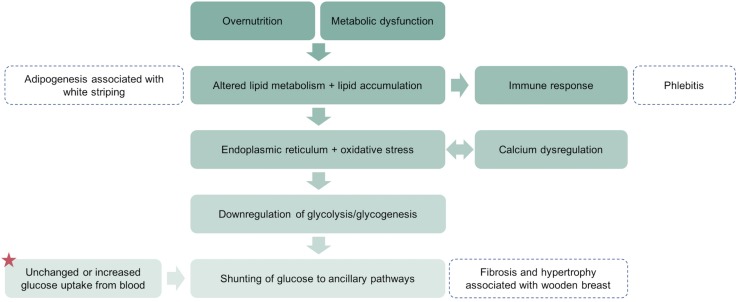
A simplified schematic representation of the proposed pathogenesis of wooden breast and white striping in the pectoralis major muscle. A key difference between wooden breast in broilers and type 2 diabetes in mammals is the dependence on insulin-independent glucose transport in skeletal muscle of chickens. This results in unchanged or increased uptake of glucose from blood (star) even when glycolysis and glycogenesis are substantially downregulated and shunting of that glucose into alternative pathways that contribute to the wooden breast phenotype.

The last half century has witnessed remarkable gains in commercial broiler production characterized primarily by rapid growth, high feed efficiency, and high breast muscle yield (Havenstein et al., 2003a, b). Alongside improvements to production traits, intense breeding programs and enhanced management practices may have elicited the emergence of several muscle disorders among fast-growing broiler strains. Myopathies such as wooden breast, white striping, spaghetti meat, and dorsal cranial myopathy significantly impact meat quality, causing substantial economic losses in the poultry industry (Zimermann et al., 2012; Kuttappan et al., 2016; Baldi et al., 2018; Zanetti et al., 2018). Speculation on the causes of these muscle disorders has focused largely on impaired oxygen supply, buildup of metabolic waste, and overstretching or compartmentalization of the muscle due to sustained rapid growth of skeletal muscle and consequent vascular marginalization (Kuttappan et al., 2013; Mudalal et al., 2015; Dalle Zotte et al., 2017; Lilburn et al., 2018). While each of these may well be a contributing factor, a critical analysis of the literature and findings in our laboratory has prompted us to submit a new hypothesis. Here we propose that dysregulation of lipid and glucose metabolism is an important underlying cause of wooden breast and related muscle disorders in commercial broiler chickens (Figure 1). Moreover, we suggest that there are substantial similarities in the mechanistic underpinnings of wooden breast in broiler chickens and type 2 diabetes in mammals.

## Lipid Accumulation in Wooden Breast Phenotype

Evidence of lipid accumulation (Papah et al., 2017) and altered lipid metabolism (Papah et al., 2018; Lake et al., 2019) early in the onset of wooden breast suggest that dysregulation of lipid metabolism is a key etiological feature of the myopathy. Lipid accumulation in the pectoralis major of wooden breast affected chickens is evident at the molecular, microscopic, and macroscopic levels. Affected breast muscle has an overall higher percent composition of fat (Soglia et al., 2016), an altered fatty acid profile (Gratta et al., 2019), and is substantially more likely than unaffected filets to exhibit signs of white striping, characterized by fatty white striations running parallel to the muscle fiber that are visible to the naked eye (Tasoniero et al., 2016; Dalle Zotte et al., 2017). Microscopic evidence of lipid accumulation and its associated pathological changes includes the presence of lipid droplets, lipogranulomas, and lipid-laden foam cells in the pectoralis major as early as the first week of age (Papah et al., 2017). At the molecular level, metabolomic profiling of 7-week-old birds shows that affected tissues have higher levels of multiple long chain fatty acids, including palmitate, palmitoleate, stearate, and oleate, as well as accumulation of various phospholipid and triglycerides catabolites (Abasht et al., 2016). Lipid accumulation is also supported by the upregulation of lipid metabolism genes in the pectoralis major of 2- and 3-week-old birds that were later diagnosed with wooden breast at 7 weeks of age (Papah et al., 2018; Lake et al., 2019). These genes include *fatty acid translocase* (*CD36*), *fatty acid binding protein 4* (*FABP4*), *lipoprotein lipase* (*LPL*), and *peroxisome proliferator-activated receptor gamma (PPARG)* among others (Lake et al., 2019). Such studies provide overwhelming evidence of altered lipid metabolism in the pectoralis major of wooden breast affected broilers starting in early phases of the disease.

Of the genes mentioned above, *LPL* stands out due to its encoded protein’s key role as a metabolic gatekeeper in terms of partitioning circulating lipids among tissues (Wang and Eckel, 2009). The *LPL* gene encodes lipoprotein lipase, an enzyme that attaches to the surface of vascular endothelial cells and serves as the rate-limiting catalyst for hydrolysis of triglycerides in two types of circulating lipoproteins – portomicrons/chylomicrons and very-low-density lipoproteins (VLDL) – providing non-esterified fatty acids and monoglycerides for use by surrounding tissues (Mead et al., 2002; Wang and Eckel, 2009). Regulation of LPL expression occurs in a tissue-specific manner and alterations to LPL levels in one tissue can affect systemic nutrient partitioning by reducing substrate availability to other tissues (Wang and Eckel, 2009). Increased expression of *LPL* is a consistent and early signal of the overprovision of lipids to the pectoralis major in affected birds. *LPL* is upregulated in 2- and 3-week-old broilers that later develop wooden breast (Papah et al., 2018; Lake et al., 2019), it has been proposed as a contributor to sex-linked differences in wooden breast incidence rate and severity (Brothers et al., 2019), and its increased expression in affected birds has been localized to the site where disease is first microscopically apparent – the endothelium of veins undergoing phlebitis (Papah and Abasht, 2019).

Lipid accumulation in the pectoralis major has been consistently linked to wooden breast, but the mechanism by which it may be involved in the myopathy has not been sufficiently explored. Papah et al. (2017) pointed out the resemblance of the lipid infiltration and phlebitis of wooden breast to atherosclerosis, although symptoms are restricted to the veins of affected broilers. It has also been suggested that increased expression of lipid metabolism genes in the early stages of wooden breast may signify a pathogenetic relationship with metabolic syndrome and type 2 diabetes in humans (Lake et al., 2019). Ectopic fat deposition and the resulting lipotoxicity, specifically in skeletal muscle, are known to be major metabolic risk factors for type 2 diabetes and related conditions (Rasouli et al., 2007), and we believe they play an important etiological role in wooden breast and similar muscle disorders. However, an important missing link between lipid accumulation and other aspects of the wooden breast phenotype is the fate of glucose in the pectoralis major.

## Lipotoxic Inhibition of Glycolysis and Glycogenesis

The pectoralis major of wooden breast affected birds exhibits signs of severely altered glucose metabolism, specifically inhibition of glycolysis. This is strongly supported by lower levels of glycolytic intermediates, such as glucose-6-phosphate and fructose-6-phosphate, and glycolytic end products, such as pyruvate and lactate, in affected birds (Abasht et al., 2016). Glycogen levels are also significantly lower (Abasht et al., 2016), which discounts the idea that increased synthesis of glycogen (glycogenesis) from glucose-6-phosphate or decreased degradation (such as glycogen storage disease) cause the reduction in glycolytic intermediates and end products. Inhibition of glycolysis is also supported by downregulation of the *glycolytic enzyme 6-phosphofructo-2-kinase* (*PFKFB3*) (Mutryn et al., 2015) as well as the isoform primarily expressed in skeletal muscle, *PFKFB1* (unpublished data), in 7-week-old affected birds. In that study, *PFKFB3* and *PFKFB1* were the only detected HIF1-dependent gene that were downregulated rather than upregulated (Mutryn et al., 2015). The suppression of glycolysis in affected birds is actually at odds with a common hypothesis that hypoxia resulting from breast muscle growth and vascular deficiency is the primary cause of wooden breast and other breast muscle disorders in modern broilers (Boerboom et al., 2018; Sihvo et al., 2018; Livingston et al., 2019a; Petracci et al., 2019). Hypoxia is widely known to stimulate glycolysis in lieu of more oxygen-demanding means of ATP production (Semenza et al., 1994). What must be determined, then, is the trigger for altered glucose metabolism as well as the fate of glucose that doesn’t undergo glycolysis in the pectoralis major.

Reduced glucose uptake from plasma is an unlikely cause of reduced glycolysis as there is no difference in plasma glucose levels between affected and unaffected birds (Livingston et al., 2019a, b) and expression of various glucose transporter genes is actually upregulated in the pectoralis major of affected birds (unpublished data). The liver has a major role in maintaining blood glucose levels, compensating for increased plasma glucose by synthesizing more hepatic glycogen (Hers, 1976). However, the livers of wooden breast affected birds also have lower glycogen content than livers of unaffected birds (Kawasaki et al., 2018). Although additional research on glucose uptake in wooden breast is required, this evidence suggests that glucose is taken up by the breast muscle, but not used for energy production or storage as fully as it is in unaffected birds.

We believe that the observed decrease in glycolytic flux is primarily a result of cellular stress responses to lipid accumulation in the pectoralis major. When lipids accumulate in cells and tissues that are not adequately equipped to metabolize or store them, such as liver and muscle, they can activate a broad range of cellular stress and immune responses such as toll-like receptor (TLR) signaling (Lee and Hwang, 2006), increased production of reactive oxygen species (ROS) (Goglia and Skulachev, 2003), endoplasmic reticulum stress, and the unfolded protein response (Volmer and Ron, 2015). These responses create deleterious effects, collectively referred to as lipotoxicity, and can destabilize metabolic functions such as glycolysis. In wooden breast, we believe oxidative stress is the key inhibitor of glycolysis based on downregulation of the glycolytic gene *PFKFB3* in affected broilers (Mutryn et al., 2015). *PFKFB3* encodes the enzyme that catalyzes the conversion of fructose-6-phosphate to fructose 2,6-bisphosphate, which itself is a potent activator of glycolysis via allosteric regulation of phosphofructokinase 1. *PFKFB3* is also considered a regulator of oxidative stress because of its ability to sense and respond to redox homeostasis, shunting glucose to the pentose phosphate pathway in response to elevated levels of ROS (Seo and Lee, 2014; Yamamoto et al., 2014).

In mammals, lipotoxic inhibition of glucose oxidation in skeletal muscle and adipose tissue is accompanied by downregulation of insulin-sensitive glucose transporter 4 (GLUT4), resulting in the insulin resistance and high plasma glucose levels that characterize type 2 diabetes (Randle et al., 1965; Groop and Ferrannini, 1993). Reduced GLUT4-mediated glucose transport is especially impactful in skeletal muscle, which is the major site of glucose uptake in the postprandial state (DeFronzo and Tripathy, 2009). This process has been demonstrated in a study of muscle-specific overexpression of LPL in transgenic mice (Kim et al., 2001). In that experiment, overexpression of muscle-specific LPL caused major metabolic changes in skeletal muscle, including a 3-fold increase in triglyceride content, an increased number of lipid droplets around the mitochondrial region, a 52% decrease in insulin-stimulated glucose uptake, a 48% decrease in glycolysis, and an 88% decrease in glycogen synthesis (Kim et al., 2001). The key difference between this mouse model of type 2 diabetes and the wooden breast phenotype is that wooden breast is not associated with a significant change in plasma glucose levels (Livingston et al., 2019a, b). However, this disparity is likely a result of differences between avian and mammalian glucose transport and insulin signaling in skeletal muscle.

## Insulin-Independent Glucose Transport in Chicken Skeletal Muscle

The polarity and size of glucose molecules prevent their transport across lipid membranes by simple diffusion (Navale and Paranjape, 2016). Two families of glucose transporters, sodium-glucose linked transporters (SGLTs) and facilitated glucose transporters (GLUTs), control the movement of glucose into and out of cells (Navale and Paranjape, 2016) and thus play an important role in glucose homeostasis and regulation of blood glucose levels. In mammals, glucose transport in skeletal muscle relies primarily on the activity of GLUT1 and GLUT4 (Klip et al., 1996). GLUT1 is preferentially restricted to the cell surface and provides insulin-independent basal levels of glucose while GLUT4 is sequestered in intracellular vesicles and rapidly translocated to the cell surface in response to insulin, exercise, or hypoxia (Klip et al., 1996; Shepherd and Kahn, 1999). Impairment of the insulin signaling cascade in skeletal muscle of mammals, which can occur in response to chronic high-fat conditions (Zierath et al., 1997), reduces the insulin-stimulated translocation of GLUT4 to the plasma membrane. Pancreatic β-cells compensate for a reduced insulin response by increasing secretion of insulin, but eventually this becomes insufficient and reduced GLUT4 translocation causes accumulation of glucose in the blood.

Avian glucose transport and insulin signaling are not as well characterized as those of mammals (Braun and Sweazea, 2008), but it is well known that chickens are naturally hyperglycemic and insulin resistant, possessing circulating insulin concentrations approximately equal to those of mammals but extremely high plasma glucose levels (Akiba et al., 1999; Tokushima et al., 2005). Insulin resistance in chickens has been partly attributed to hyperactivity of insulin receptor signaling in skeletal muscle, where phosphatidylinositol 3-kinase (PI3K) activity, a key component of the insulin signaling pathway, was found to be 30 times higher in chickens than in rats (Dupont et al., 2004). In fact, insulin privation via immuno-neutralization was found to have no effect on PI3K activity in chicken skeletal muscle, although it resulted in altered expression of major metabolic transcription factors in both the liver and skeletal muscle (Godet et al., 2008). Another major contributor is perhaps the intrinsic lack of any GLUT4 homologs in the chicken genome and resulting predominance of insulin-independent glucose transport. An attempt to detect GLUT4 homologs in various tissues of broiler chickens found that GLUT4 cDNA was completely undetectable in any of the 19 chicken tissues that were tested, which included the pectoralis major (Seki et al., 2003). The intrinsic lack of GLUT4 and low expression levels of other common glucose transporters (GLUT1, GLUT2, GLUT3, and GLUT8) in skeletal muscle of broilers (Kono et al., 2005) suggests that glucose transport is regulated differently in chickens than it is in mammals and may primarily be insulin-independent.

The reliance on insulin-independent glucose transporters in chicken skeletal muscle may explain why there are no mammalian skeletal muscle disorders that are equivalent to wooden breast. Rather, the wooden breast phenotype shares striking similarities with various complications of type 2 diabetes in smooth muscle and cardiac muscle, such as atherosclerosis, diabetic cardiomyopathy and myocardial fibrosis, diabetic nephropathy, pulmonary fibrosis, diabetic retinopathy, and non-alcoholic fatty liver disease. These diseases frequently involve lipid accumulation, inflammation, oxidative stress, calcium dysregulation, endoplasmic reticulum stress, hypoxia, hypertrophy, and fibrosis – features that have also been well-documented in wooden breast. The greater role of insulin-independent glucose transport in cardiac and smooth muscle allows glucose uptake even when glycolysis and glycogenesis are impaired. The importance of insulin-insensitive glucose transport in development of diabetic retinopathy (Kumagai, 1999), atherosclerosis (Wall et al., 2018), diabetic nephropathy (Brosius and Heilig, 2005), pulmonary fibrosis (Cho et al., 2017), diabetic myocardial fibrosis (Asbun and Villarreal, 2006; Gorski et al., 2019) and non-alcoholic fatty liver disease (Nanji et al., 1995) has been demonstrated and is in support of our hypothesis that wooden breast shares substantial etiological factors with type 2 diabetes in mammals.

## Pathological Shunting of Glucose to Ancillary Pathways

The suppression of glycolysis and glycogenesis concurrent with unchanged or increased uptake of glucose from blood results in increased flux of glucose through alternative metabolic pathways (Fantus et al., 2006), including the pentose phosphate, glucuronic acid, hexosamine biosynthesis, and aldose reductase (polyol) pathways. There is evidence that all of these pathways are upregulated in affected birds (Abasht et al., 2016; Papah et al., 2018).

Glucose that is phosphorylated to glucose-6-phosphate can directly enter both the pentose phosphate pathway and the glucuronic acid pathway. In addition to generating carbon skeletons and ribose 5-phosphate, a precursor to nucleotide synthesis, the pentose phosphate pathway is a major source of NADPH, the main reductant that drives free radical detoxification and anabolic growth (Kruger and Von Schaewen, 2003). Accumulation of pentose phosphate pathway intermediates 6-phosphogluconate and sedoheptulose 7-phosphate support the upregulation of this pathway in 7-week-old affected birds (Abasht et al., 2016). Glucose-6-phosphate is also utilized to produce glucuronic acid, a precursor to ascorbic acid and a building block of proteoglycans, glycosaminoglycans, and glycolipids. Elevated levels of ascorbate and UDP-glucuronate in affected birds suggests that this pathway is upregulated as well (Abasht et al., 2016).

Glucose-6-phosphate can also be converted to fructose-6-phosphate and consumed in the hexosamine biosynthesis pathway. The hexosamine biosynthesis pathway is responsible for production of uridine diphosphate N-acetylglucosamine (UDP-GlcNAc), a nucleotide sugar and coenzyme used for protein glycosylation and the synthesis of glycosaminoglycans, proteoglycans, and glycolipids (Fantus et al., 2006). The gene encoding this pathway’s rate-limiting enzyme, glutamine-fructose-6-phosphate transaminase 2 (GFPT2), was found to be upregulated in 3-week-old broilers affected by wooden breast (Papah et al., 2018). In fact, three of the four genes involved in the hexosamine biosynthesis pathway – *GFPT2*, *phosphoglucomutase 3* (*PGM3*), and *UDP-N-acteylglucosamine pyrophosphorylase 1* (*UAP1*) – show increased expression in the pectoralis major of high-feed-efficiency broilers, which are more susceptible to wooden breast than those with low feed efficiency (Abasht et al., 2019). In agreement, affected birds have higher levels of hexosamine biosynthesis pathway intermediates isobar UDP-acetylglucosamine and UDP-acetylgalactosamine at 7 weeks of age (Abasht et al., 2016). Increased production of proteoglycans is further supported by Clark and Velleman (2017), who found greater RNA expression of decorin in the pectoralis major of affected birds. The protein encoded by this gene is a small leucine-rich proteoglycan that regulates collagen cross-linking, although it is unclear if increased collagen cross-linking is a universal feature of the wooden breast phenotype (Velleman et al., 2017; Baldi et al., 2019). Proteoglycans and glycosaminoglycans are important components of the extracellular environment and increased production of them is associated with extensive remodeling of the extracellular matrix.

Excess glucose is also consumed in the polyol pathway, a two-step process that converts glucose first to sorbitol and then to fructose (Yan, 2018). Upregulation of the polyol pathway, specifically its first step, in wooden breast and white striping is supported by an accumulation of sorbitol in the pectoralis major (Abasht et al., 2016; Boerboom et al., 2018). One notable effect of polyol pathway stimulation is the induction of collagen synthesis (Bleyer et al., 1994; Ha et al., 1997) due at least in part to transcriptional activation of transforming growth factor-β (TGF-β) (Ishii et al., 1998; Han et al., 1999; Tokudome et al., 2004). All three isoforms of TGF-β are considered important regulators of inflammation, extracellular matrix protein deposition, and fibrosis, and altered activity of TGF-β proteins is known to contribute to various fibroproliferative disorders in humans (Pohlers et al., 2009). Increased expression of *transforming growth factor* β *3* (*TGFB3*) in the pectoralis major muscle of affected birds at 7 weeks (Mutryn et al., 2015) may represent a link between altered glucose metabolism and increased collagen production in wooden breast, the latter of which is widely considered to be an important feature of the myopathy contributing to the characteristic firmness of the pectoralis major (Sihvo et al., 2014; Soglia et al., 2016; Papah et al., 2017).

Finally, glucose can be used in the production of advanced glycation end products (Fantus et al., 2006). Advanced glycation end products are proteins or lipids that become glycated as a result of exposure to sugars and can have numerous pathological effects, such as induction of cytokine production, increased vascular permeability and inflammation, inhibition of vascular dilation, and enhanced oxidative stress (Basta et al., 2004). These effects are consistent with current knowledge of wooden breast, although altered production of advanced glycation end products has not yet been reported.

**FIGURE 2:**
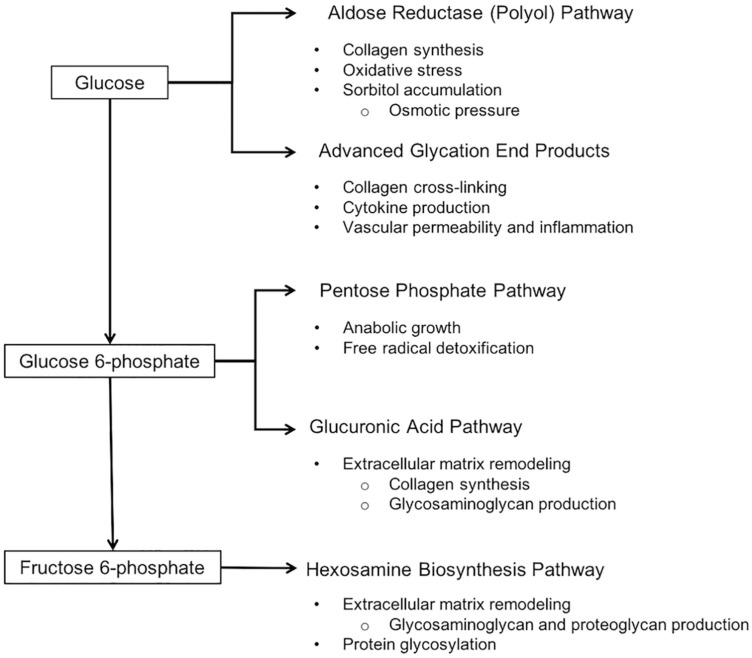
Altered carbohydrate metabolism in wooden breast and relevant effects of each pathway. Wooden breast is associated with a reduced flux of glucose through glycolytic and glycogenic pathways and shunting of glucose into ancillary pathways, including the aldose reductase, pentose phosphate, glucuronic acid, and hexosamine biosynthesis pathways. It has not yet been demonstrated whether or not wooden breast also involves increased synthesis of advanced glycation end products.

Increased flux of glucose through these alternative pathways (Figure 2) is a pathological manifestation of altered glucose utilization, likely resulting from reduced glycolysis and glycogenesis alongside unchanged or increased import of glucose from the blood. This process is often referred to as glucose toxicity and is considered a key component of pancreatic β-cell dysfunction, insulin resistance, and chronic complications of diabetes such as diabetic neuropathy, retinopathy, and nephropathy in mammals.

## Disruption of Redox Homeostasis and Oxidative Stress

Oxidative stress occurs when the production and accumulation of ROS become unbalanced with an organism’s ability to detoxify reactive intermediates and can lead to cell and tissue damage. Broiler chickens show signs of greater oxidative stress in the pectoralis major than layers (Zahoor et al., 2017) and broilers with high feed efficiency have greater oxidative stress response in the breast muscle compared to those with low feed efficiency (Zhou et al., 2015). In wooden breast, affected broilers have metabolite profiles indicative of altered redox homeostasis involving higher free radical exposure than unaffected birds (Abasht et al., 2016). Similarly, various genes involved in oxidative stress response are upregulated in wooden breast birds at both 3 and 7 weeks of age (Abasht et al., 2015; Mutryn et al., 2015; Papah et al., 2018).

As previously discussed, lipotoxicity is likely a major contributor to oxidative stress in the early stages of wooden breast. Fatty acids are particularly prone to ROS-induced oxidative damage via a process called lipid peroxidation, which proceeds in an aggressively self-propagating chain reaction that can induce damage to proteins and DNA (Goglia and Skulachev, 2003; Schrauwen, 2007). When the supply of fatty acids overwhelms the storage and oxidative capacity of cells, which is more likely in mitochondria-scarce, type IIB pectoralis major muscle fibers, they accumulate in the cell and around the mitochondria where they can have extremely damaging effects (Schrauwen and Hesselink, 2004; Anderson and Neufer, 2006). Fatty acid transport into the mitochondria is generally regulated by the enzyme carnitine palmitoyltransferase 1 (Schrauwen, 2007). Lower levels of free carnitine in the pectoralis major of affected birds (Abasht et al., 2016) may reflect increased transport of long-chain fatty acids into the mitochondria by carnitine palmitoyltransferase 1. Fatty acids and fatty acid derivatives inside the mitochondrial matrix are extremely vulnerable to ROS-induced lipid peroxidation and can cause substantial mitochondrial damage due to the complex nature of the matrix space (Goglia and Skulachev, 2003). Oxidative damage to the mitochondria due to fatty acid accumulation may be attenuated by mitochondrial energy uncoupling, mediated largely by uncoupling protein 3 (UCP3), an enzyme activated by lipid peroxides (Goglia and Skulachev, 2003; Rousset et al., 2004; Schrauwen and Hesselink, 2004). However, *UCP3* was found to be downregulated in wooden breast affected birds at 3 weeks of age (Papah et al., 2018), suggesting an impaired uncoupling response. High coupling efficiency in an environment with severe lipid accumulation would directly contribute to ROS production and lipid peroxidation and also allow for increased mitochondrial damage by ROS inside the mitochondrial matrix.

After initial derangement of redox homeostasis, multiple factors can contribute to a positive feedback loop of oxidative stress. Most ROS are generated as normal by-products during mitochondrial electron transport, specifically at respiratory complexes I and III of the oxidative phosphorylation pathway (Rousset et al., 2004), and are prevented from reaching damaging levels by various cellular defenses such as superoxide dismutase and glutathione peroxidase (Anderson and Neufer, 2006). Potential genetic variation in antioxidant response may be a key contributor to wooden breast susceptibility in broilers, as altered redox homeostasis can inhibit the activity of anti-oxidant enzymes and increase ROS production at mitochondrial respiratory complex I (Yabe-Nishimura, 1998; Chung, 2004; Yan, 2018). In diabetes, the polyol pathway is believed to play a critical role in oxidative stress and vascular damage due to its derangement of redox homeostasis (Yabe-Nishimura, 1998; Chung, 2004; Yan, 2018). The first of two reactions in the pathway is conversion of glucose and NADPH to sorbitol and NADP+ by aldose reductase and is suggested to be upregulated in the pectoralis major of affected birds based on the accumulation of sorbitol (Abasht et al., 2016; Boerboom et al., 2018). Mutryn et al. (2015) proposed NADPH-oxidase activity as another contributor to altered NADP + /NADPH homeostasis related to inflammatory and immune responses. The consumption of NADPH by aldose reductase and NADPH-oxidase reduces the activity of other NADPH-dependent enzymes such as glutathione reductase, an important anti-oxidative enzyme, and nitric oxide synthase, which produces nitric oxide from L-arginine (Yabe-Nishimura, 1998). Nitric oxide is a soluble gas produced by endothelial cells that has many functions, including regulation of vascular homeostasis, vasodilation, angiogenesis, endothelial cell growth, and protection of vessels from injury (Tousoulis et al., 2012).

Disruption of NAD+/NADH homeostasis can also increase ROS production, wherein overproduction of the electron donor NADH increases activity of NADH-dependent mitochondrial respiratory complex I (Hirst et al., 2008). The conversion of sorbitol and NAD+ to fructose and NADH by sorbitol dehydrogenase (SORD) in the second reaction of the polyol pathway is one potential contributor to this imbalance; however, this is unlikely in wooden breast as decreased *SORD* expression (unpublished data) and greater accumulation of sorbitol (Abasht et al., 2016) in affected birds provide testimony for reduced SORD activity. Rather, potentially higher levels of NADH in wooden breast may be partly attributed to lower activity of the glycerol-3-phosphate shuttle (Abasht et al., 2019) or lower activity of lactate dehydrogenase. An imbalance of cytosolic NAD+/NADH would not only increase ROS production but could also limit NAD+ supply to key metabolic enzymes required for sustaining glycolysis and the citric acid cycle. Free NAD+ is a cofactor for glyceraldehyde 3-phosphate dehydrogenase and oxoglutarate dehydrogenase, both of which are downregulated in affected birds according to differential expression analysis (Abasht et al., 2015; Papah et al., 2018). Free NAD+ is also a cofactor for the pyruvate dehydrogenase complex which serves as a link between glycolysis and the citric acid cycle (Yan, 2018).

## Calcium Cycling Abnormalities

Maintenance of intracellular Ca^2+^ pools is fundamental to generating the Ca^2+^ signals required for numerous cellular processes (Berridge et al., 2003). The pectoralis major of wooden breast affected birds exhibits upregulation of genes encoding both parvalbumin and sarcoplasmic/endoplasmic reticulum calcium ATPase (SERCA) 2 (Mutryn et al., 2015) as well as increased abundance of SERCA protein (Soglia et al., 2016). Both proteins are involved in sequestering calcium and their upregulation constitutes evidence of a compensatory response to increased intracellular calcium in muscle cells (Mutryn et al., 2015). Parvalbumin is a Ca^2+^-binding protein that functions as a calcium buffer and SERCAs are intracellular pumps located in the sarcoplasmic/endoplasmic reticulum (SR) membranes that use ATP to translocate Ca^2+^ from the cytoplasm to the SR lumen (Berridge et al., 2003). Evidence of dysregulated calcium homeostasis and impaired excitation-contraction coupling is also present in 2- and 3-week-old broilers, prior to manifestation of wooden breast phenotype at market age (Papah et al., 2018; Lake et al., 2019). Increased levels of calcium in the pectoralis major of birds affected by white striping, wooden breast, or both (Sandercock et al., 2009; Tasoniero et al., 2016; Zambonelli et al., 2016) suggests that myocellular uptake of Ca^2+^ from extracellular spaces is affected in addition to dysregulation of intracellular calcium pools.

It is unclear what provides the primary stimulus for calcium dysregulation in wooden breast and, unfortunately, there is no consensus regarding a single cause of altered calcium cycling in diabetes despite extensive research. However, one popular hypothesis contends that chronic exposure to excessive nutrients, specifically glucose and lipids, in tissues unequipped to fully metabolize, store, or dispose of them can initiate SR and mitochondrial stress that result in disruption of calcium homeostasis (Arruda and Hotamisligil, 2015). At least three mechanisms can aid in understanding this process: (1) fatty acids and ROS stimulate release of Ca^2+^ from the SR and inhibit removal of Ca^2+^ from the cytosol, (2) formation of the mitochondrial permeability transition pore and mitochondrial depolarization cause release of Ca^2+^ from the mitochondria, and (3) depolarization of the plasma membrane causes an influx of Ca^2+^ from extracellular spaces. The SR lumen serves as the most important Ca^2+^ store in the cell (Arruda and Hotamisligil, 2015), but an excess of fatty acids, fatty acid derivatives, and ROS can cause release of Ca^2+^ from the SR lumen by activating ryanodine and inositol trisphosphate (IP_3_)-sensitive calcium channels (Cheah, 1981; Ursini et al., 2004; Berezhnov et al., 2008; Wei et al., 2009). Certain types of fatty acids can also inhibit SERCA activity, preventing calcium removal from the cytosol (Berezhnov et al., 2008; Fu et al., 2011). Fluxes in intracellular calcium can be buffered by increased calcium uptake in the mitochondria, where Ca^2+^ can elevate ATP production (Berridge et al., 2003). However, excess mitochondrial Ca^2+^ can sensitize the mitochondrial permeability transition pore, leading to membrane depolarization and the rapid release of Ca^2+^ into the cytosol (Gunter et al., 1994; Berezhnov et al., 2008). High concentrations of long-chain fatty acids could also stimulate formation of the mitochondrial permeability transition pore (Penzo et al., 2002).

Calcium released from internal stores in the SR and mitochondria cannot explain why the pectoralis major of wooden breast birds has an increased percent composition of Ca^2+^. Dysregulation of cation homeostasis that extends beyond Ca^2+^ in affected birds provides evidence that an influx of Ca^2+^ into the cytoplasm from extracellular spaces involves cell membrane depolarization. Specifically, affected birds show higher sodium and lower magnesium and phosphorus in the pectoralis major as well as higher blood potassium levels (Zambonelli et al., 2016; Livingston et al., 2019b). At resting condition, the concentration of K^+^ is higher inside the cell while the concentration of Na^+^ and Ca^2+^ is higher outside the cell (Bahar et al., 2016), but the opening of voltage-gated calcium and sodium channels during membrane depolarization results in the rapid influx of Ca^2+^ and Na^+^ from extracellular spaces (Bahar et al., 2016). The subsequent efflux of K^+^ through voltage-dependent and voltage-independent channels allows repolarization of the membrane (Bahar et al., 2016). Calcium influx in wooden breast might be induced by certain types of fatty acids (Huang et al., 2006), by oxidative damage to the cell membrane (Ursini et al., 2004), or by the gradient-dependent activity of Na^+^/Ca^2+^ exchangers that exchange three moles of Na^+^ for one mole of Ca^2+^ (Berridge et al., 2003). Opening of voltage gated calcium channels can exacerbate the release of calcium from endoplasmic reticulum stores in a process called calcium-induced calcium release (Endo, 2009).

One notable effect of altered calcium cycling in skeletal muscle, specifically of increased ATP-dependent SERCA activity, is a substantial increase in oxygen consumption and concurrent elevation of resting metabolic rate (Smith et al., 2013). Disruption of calcium homeostasis is potentially a major contributor to the venous hypercapnia, venous hypoxemia, and muscular hypoxia of the pectoralis major that have been documented in wooden breast affected broilers (Mutryn et al., 2015; Livingston et al., 2019a, b). Other effects of calcium dysregulation include mitochondrial damage (Orrenius et al., 2015), aberrant excitation/contraction signaling (Berridge et al., 2003), chronic SR stress (Fu et al., 2011), stimulation of skeletal muscle growth (Ermak and Davies, 2001), calcium-dependent proteolysis (Yin et al., 2013), and ultimately cell death (Hajnóczky et al., 2006). As a major site of post-translational protein modification, the SR is crucial to producing functional proteins. Calcium-dependent proteolysis and SR stress could contribute to the lower protein content and lower water holding capacity of wooden breast filets due to their effects on protein degradation and protein production.

Dysregulation of calcium signaling can also induce expression of genes encoding myoglobin and slow muscle fiber type proteins, a feature of wooden breast discovered through differential expression analysis (Mutryn et al., 2015) and confirmed with RNA *in situ* hybridization (RNA ISH) (Papah and Abasht, 2019). Expression of myoglobin and other muscle-specific genes is regulated by a synergistic interaction between transcription factors in the nuclear factor of activated t cells (NFAT) and myocyte enhancer factor-2 (MEF-2) families (Chin et al., 1998). Transcriptional activation of NFAT and MEF-2 is mediated by calcineurin, a calcium-activated phosphatase, such that calcium fluxes and intracellular concentrations will ultimately determine the fiber type composition within a specific skeletal muscle (Chin et al., 1998). Lipid supplementation and hypoxic conditions have also been demonstrated to stimulate myoglobin expression, possibly through calcium-independent pathways (De Miranda et al., 2012).

## Venous Inflammation and Vascular Permeability

Venous and perivascular inflammation and lipid accumulation are the first microscopic signs of wooden breast, potentially contributing to the edema, petechial hemorrhages, and tissue damage that are macroscopically apparent in late stages of the disease (Papah et al., 2017). It has been noted that these symptoms resemble atherosclerosis in humans (Papah et al., 2017), a condition closely linked to diabetes mellitus that consists of chronic inflammation induced by excessive lipid accumulation (Li et al., 2014). The upregulation of genes associated with vascular disease in 3-week-old broilers that later develop wooden breast (Papah et al., 2018) corroborates this hypothesis, but fails to explain why vascular inflammation is limited to veins. Recent work using RNA ISH to localize expression of a selection of genes in affected pectoralis major muscle provides an important clue. In that study, higher LPL expression was found in the veins of affected breast muscle and not the arteries (Papah and Abasht, 2019), suggesting a causal link between venous and perivascular lipid accumulation and the inflammatory response. This connection between increased LPL activity and inflammation has been thoroughly studied in the context of atherosclerosis.

The participation of LPL in the pathogenesis of atherosclerosis is two-fold: it mediates both the increased hydrolysis of lipoprotein triglycerides as well as the retention of lipoprotein remnants. High LPL activity increases local hydrolysis of triglycerides in portomicrons and VLDL, which produces free fatty acids, portomicron remnants, intermediate-density lipoproteins, and low-density lipoproteins (Alvarenga et al., 2011). Elevated levels of fatty acids and lipoprotein remnants cause damage to vessels as they trigger an inflammatory response, induce endothelial cell apoptosis, and increase endothelial permeability (Toborek et al., 2002; Eiselein et al., 2007; Rocha et al., 2016). A critical element of the inflammatory response caused by fatty acids is the activation of TLRs in the presence of high glucose (Lee and Hwang, 2006; Dasu and Jialal, 2010). TLR activation initiates an inflammatory cascade that includes the release of pro-inflammatory cytokines and cell adhesion molecules as well as T cell activation and the rapid differentiation of monocytes into macrophages (Krutzik et al., 2005). In wooden breast affected birds, *toll-like receptor 2 type 2 precursor* (*TLR2-2*) was one of the top 30 genes identified as a biomarker of wooden breast severity, with significant elevation in moderately affected birds compared to unaffected or severely affected birds (Abasht et al., 2015). The author suggested that this expression pattern may be indicative of the progression of the disease involving regulation by some negative feedback mechanism. If disease severity and disease progression are considered more or less equivalent, a similar pattern can be seen with lipid metabolism genes, which are generally upregulated in 2- and 3-week-old birds but relatively unchanged or downregulated in 7-week-old birds (Mutryn et al., 2015; Papah et al., 2018; Papah and Abasht, 2019). Due to the apparent coupling of lipid metabolism and venous inflammation, negative feedback regulation of lipid metabolism could be associated with a reduction in the innate immune response.

Endothelial cell damage and apoptosis due to increased lipoprotein metabolism increases the permeability of the endothelium and triggers leukocyte adhesion and transmigration into the vessel wall (Aghajanian et al., 2008). Lipoprotein remnants, which normally enter back into circulation to be cleared by the liver (Alvarenga et al., 2011), can then diffuse into the tunica intima where LPL mediates binding between remnant particles and proteoglycans in the sub-endothelial extracellular matrix (Olin et al., 1999). The subsequent activation of extracellular matrix proteins such as matrix metalloproteinases can elicit remodeling of the extracellular matrix (Amin et al., 2017), a process that is a well-substantiated in the wooden breast pathogenesis (Mutryn et al., 2015; Papah et al., 2018). LPL-mediated bridging normally occurs between lipoprotein remnants and heparan sulfate proteoglycans on cell surfaces before lipoproteins undergo hydrolysis (Olin et al., 1999). Increased retention of lipoprotein remnants in the extracellular matrix and on cell surfaces increases the likelihood that they will be modified (e.g., oxidized) or taken up by scavenging macrophages to form foam cells, triggering further inflammatory reactions (Botham et al., 2007). The pro-atherosclerotic role of both active and inactive LPL has been demonstrated in mice (Wang et al., 2007), suggesting that both the hydrolysis and bridging functions of LPL are important.

## Broiler Selection and Wooden Breast

Consolidation of poultry breeding and intense selection for production traits have undoubtedly played an oversized role in the rise of muscle disorders such as wooden breast, white striping, and spaghetti meat among commercial broilers. Even without completely understanding the genetic architectures underlying these traits, it is possible to speculate how selection for specific performance metrics might increase the predisposition of meat-type chickens to these myopathies. Much of this speculation can be framed in the context of supply and demand of nutrients in the pectoralis major muscle. Juvenile growth rate, which has increased substantially since the 1950s independent of improvements to feed formulation (Havenstein et al., 2003b) and which is correlated with wooden breast severity, represents a major component of the supply side of this equation. The rapid growth rate of modern broilers reflects increased delivery of nutrients to the whole body, stemming from some combination of greater appetite, suppressed satiety (Barbato, 1994), as well as improved digestion and absorption in the digestive system (Smith et al., 1990).

Selection for feed efficiency has also raised digestive and absorptive capacity but may not increase the overall supply of nutrients to the body as increased energy absorption from feed is at least partly offset by reduced feed consumption. A larger effect of using feed efficiency as a major selection criterion relates to its correlation with fat distribution and blood lipid levels (Griffin et al., 1989; Zhuo et al., 2015). Unlike selection for juvenile growth rate, which is accompanied by increased fat deposition in adipose tissue depots, selection for increased feed efficiency is accompanied by reduced fat deposition in adipose tissue depots (Abasht et al., 2020). This reflects altered nutrient partitioning in high feed efficiency birds which is likely multifaceted, involving changes to metabolic processes in several organs such as reduced adipogenesis in abdominal fat, reduced lipogenesis in the liver, and increased lipoprotein triglyceride hydrolysis in skeletal muscle. Regardless of the specific biological mechanisms, there is evidence that improvements to such performance traits are associated with a shifting of fat metabolism away from the liver and normal adipose depots toward skeletal muscle (Griffin et al., 1989; Larkina et al., 2010; Zhuo et al., 2015). In commercial broiler chickens, selection for large breast muscle partitions more nutrients toward the pectoralis major, which may be particularly susceptible to metabolic perturbations due to its primary composition of type IIB glycolytic muscle fibers (Anderson and Neufer, 2006).

It should be noted, though, that wooden breast does not correlate perfectly with performance traits like growth rate, feed efficiency, abdominal fat percentage, or breast muscle yield, suggesting the existence of etiological factors that may be unrelated to nutrient partitioning. For example, a mutation that affects coupling of lipid or glucose uptake and utilization in the pectoralis major might constitute the spark on the proverbial fire of nutrient accumulation and toxicity. Lake et al. (2019) proposed a potential mechanism by which this type of mutation, seemingly unrelated to performance traits, might inadvertently become the target of extreme selection pressure. Organ hypertrophy is a common symptom of chronic diabetes complications, including diabetic cardiomyopathy, nephropathy, and non-alcoholic fatty liver disease. Assuming a similar pathogenesis in wooden breast, it is possible that selection for high breast muscle yield would directly select for pathological hypertrophy symptomatic of the wooden breast phenotype. This inherent conflation of desired performance traits with muscle disorder symptoms suggests that the only long-term solution to myopathies like wooden breast and white striping involves selection against their causal variants.

## Concluding Remarks

We believe that the wooden breast phenotype in commercial broilers is a manifestation of lipotoxicity and glucotoxicity resulting from the chronic oversupply of both lipids and carbohydrates to the pectoralis major and also the disruption of normal lipid and glucose metabolism. Dependence on insulin-independent glucose transport in the skeletal muscle of chickens causes lipid accumulation in the pectoralis major to be accompanied by unchanged or increased uptake of glucose, causing metabolic and structural alterations that closely resemble complications of diabetes in smooth and cardiac muscle of mammals. In addition to improving our understanding of the etiology and pathogenesis of wooden breast and related myopathies, this hypothesis supports the use of these muscle disorders as models of human metabolic diseases.

## Data Availability Statement

The datasets generated for this study are available on request to the corresponding author.

## Author Contributions

JL wrote the original draft of the manuscript. BA provided indispensable guidance, expertise, and unpublished data from previous research. All authors contributed to critical feedback and helped to shape the research, analysis, and manuscript.

## Conflict of Interest

The authors declare that the research was conducted in the absence of any commercial or financial relationships that could be construed as a potential conflict of interest.
